# Positional vertigo ‒ beyond benign paroxysmal positional vertigo: Case report^☆^

**DOI:** 10.1016/j.bjorl.2024.101406

**Published:** 2024-02-21

**Authors:** Lucas Scatolin Partezani, Raquel Mezzalira, Luis Augusto Guedes de Mello Dias, Daniela Akemi Souza Saito, Marina Saes Rays, Durval de Paula Chagas Neto

**Affiliations:** Instituto Penido Burnier, Campinas, SP, Brazil

## Introduction

This report explores the diﬀ ;erential diagnosis of positional vertigo in a patient with benign paroxysmal positional vertigo-like symptoms but with unusual progression, necessitating investigation into less common pathologies.

## Case report

Our patient, a 52-year-old woman, complained of spinning vertigo episodes triggered when she lay down and turned her head to the right, causing her to feel nauseous. These episodes lasted less than 10 s, and recovery was immediate upon returning to an upright position. No associated symptoms such as headache, neck pain, or visual disturbances were reported.

On physical examination, there were no spontaneous or gaze nystagmus. Head Impulse Test, Head Shaking, Skew Deviation Test, Romberg, and Fukuda were normal.

Benign Paroxysmal Positional Vertigo (BPPV) was initially suspected. The Dix Hallpike maneuver revealed torsional counterclockwise nystagmus with delayed onset lasting less than one minute when the head was positioned to the right, suggesting involvement of the right posterior semicircular canal. When sitting up, there was no nystagmus.

The Epley maneuver was performed. However, her symptoms persisted after a week, with the same nystagmus pattern during the Dix-Hallpike maneuver.

The Epley maneuver was then repeated. After yet another week, the patient still experienced dizziness and showed the same alterations during the Dix-Hallpike maneuver. Both maneuvers were performed with the help of video-oculography.

Audiometry and tympanometry were normal.

**Figure 1:**
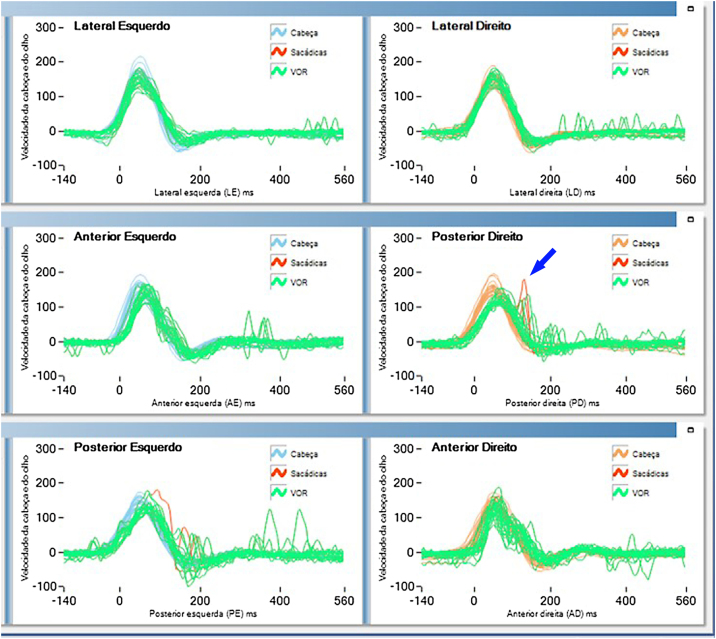
Covert saccades recorded in right semicircular posterior canal.

Further investigation using the video head impulse test revealed covert saccades in the right semicircular posterior canal (Fig. 1).

All laboratory test results, including lipidic profile, glycemic profile, thyroid profile, calcium, parathormone, vitamin D, B12 dosage, and hemogram, were normal. The patient tested negative for syphilis.

**Figure 2:**
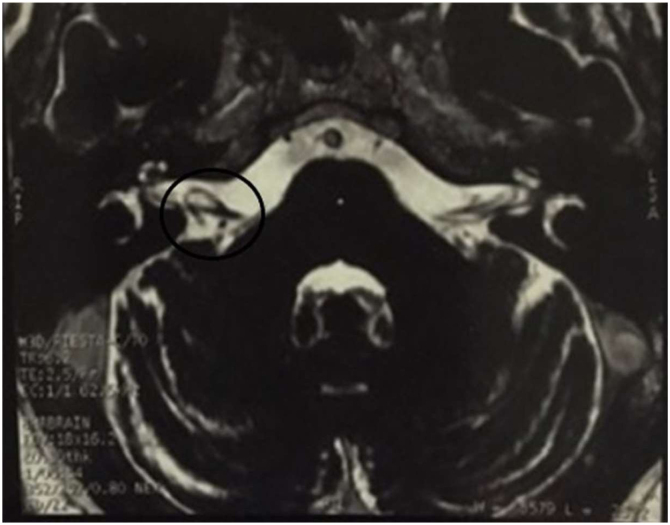
Vascular loop within the right inner auditory canal.

Magnetic Resonance Imaging (MRI) showed a vascular loop within the right inner auditory canal (Fig. 2).

Considering the possibility of vestibular paroxysmia, a therapeutic test with Gabapentin 300 mg per day was initiated. The patient’s condition improved significantly by the third day of treatment, and she remained asymptomatic for two years under observation.

## Discussion

Vertigo is a common complaint, aﬀ ;ecting 42% of adults with an estimated 8% resulting from vestibular causes in the city of São Paulo.^1^ Positional vertigo, particularly BPPV, is the most prevalent.^1^

BPPV is characterized by brief vertigo episodes when the patient lies down and turns head to a specific position.^2^

Diagnosis requires identifying characteristic nystagmus during canal evaluation.^2^ However, if nystagmus shows atypical features, or symptoms persist despite correct maneuvers, imaging examination is essential to evaluate the central nervous system.^2^

Common diﬀ ;erential diagnoses include central positional vertigo due to migraines or cerebellum and brainstem structural issues.^2^

In the discussed case, a torsional counterclockwise nystagmus was observed during the Dix-Hallpike maneuver on the right side, suggesting a compromised posterior semicircular canal. However, the lack of response to initial treatment led to further diﬀ ;erential diagnosis. Subsequent MRI revealed an Anterior Inferior Cerebellar Artery (AICA) encroaching into the internal auditory canal (Fig. 2).

When BPPV-like symptoms persist post-treatment, thorough exploration of alternative diagnoses is crucial for accurate identification and effective treatment.^2^ As the nystagmus suggested posterior semicircular canal impairment, a video head impulse was performed, revealing a covert saccade in posterior semicircular canal (Fig. 1).

In the vestibulo-ocular reflex’s central circuit, the posterior semicircular canal uniquely lacks an alternative route. It exclusively traverses the medial longitudinal fasciculus, connecting the contralateral inferior rectus and ipsilateral superior oblique muscles, resulting in counterclockwise and clockwise rotatory horizontal ocular movements, depending on the stimulated side.^3^

In this case, covert saccades in the posterior semicircular canal suggested chronic impairment, likely due to a vascular loop affecting the vestibular nerve.^3^ This explained the observed ocular movements in the Dix Hallpike maneuver. The lack of improvement post-repositioning maneuvers indicates a neural conflict in the cerebellopontine angle, not BPPV.

Vestibular paroxysmia presents as spontaneous recurring vertigo episodes.^4^ Some patients may experience episodes triggered by head movement while others may exhibit auditory symptoms, or vertigo precipitated by postural changes during crises.^4^

Diagnosis is clinical, based on brief recurrent vertigo episodes and response to carbamazepine and oxcarbazepine.^4^ Gabapentin is recommended by some authors for paroxysmia treatment due to its tolerability.^5^

Gabapentin was administered due to its ease of adaptation and minimal side effects. The initial dosage starts at 300 mg and can be increased up to 900 mg a day.^5^ The regimen was maintained for six months with gradual dosage reduction. The positive response to gabapentin confirmed the diagnosis hypothesis of vestibular paroxysmia.^4^

In addition to vestibular paroxysmia, several other conditions require consideration in the differential diagnosis of positional vertigo. These include Vestibular Migraine, Vestibular Artery Occlusion Syndrome, Dizziness of Cervical Origin, Tumors and Cysts in the Cerebellopontine angle, and Orthostatic Intolerance. Each condition presents unique characteristics and requires specific diagnostic and treatment approaches.^2^

## Conclusion

Nystagmus during positional maneuvers is not exclusively indicative of BPPV. If a patient with BPPV-like symptoms does not improve after the initial standard treatment, a comprehensive exploration of diﬀ ;erential diagnoses by means of complementary examinations is necessary in order to determine the course of action.

## Conflicts of interest

The authors declare no conflicts of interest.
